# “They’re not used to people asking what they need”: work processes and care practices in feminist abortion accompaniment – a qualitative study

**DOI:** 10.1080/26410397.2026.2676395

**Published:** 2026-05-20

**Authors:** Nathália Machado Cardoso, Naomi Braine, Ana Flávia Pires Lucas D’Oliveira

**Affiliations:** a Doctoral Student in Collective Health, Department of Preventive Medicine, Faculty of Medicine, University of São Paulo, São Paulo, Brazil.; b Professor, Department of Sociology, Brooklyn College, City University of New York (CUNY), New York, NY, USA; c Professor, Department of Preventive Medicine, Faculty of Medicine, University of São Paulo, São Paulo, Brazil

**Keywords:** abortion, accompaniment, activism, work process, Latin America, feminism, reproductive rights

## Abstract

This qualitative study examines the work process of feminist activists who provide accompaniment for self-managed abortions in a major urban centre in Brazil, where abortion remains criminalised in most circumstances. Based on in-depth interviews with eight accompaniers, and drawing on Brazilian collective health and feminist ethics of care, it analyses how they integrate technique, ethics, political commitment, and social awareness, thereby transforming accompaniment into a genuine practice of care. Findings show that accompaniment recognises those cared for as subjects with knowledge, desires, and evolving needs, rather than as passive recipients. Care is organised collectively but always tailored and responsive to the needs of each person, in an interactive, critical, and reflexive manner. In doing so, accompaniers combine technical-scientific knowledge with experiential knowledge built through prior care and exchanges within trusted networks. By engaging across all stages of the process, and guided by a project whose ultimate purpose is to make abortion a good experience for those accompanied, accompaniers prevent the fragmentation and alienation of care work. Accompaniment thus emerges as a model of care grounded in radical needs: needs that arise within existing social conditions but cannot be fully satisfied within them. Once recognised and collectively articulated, these needs expose the limits of the existing order and become a source of pressure for social transformation. The high personal cost of this activism, marked by overload, insecurity, and invisibility, challenges its sustainability and expansion. By describing its constitutive elements, this study broadens understanding of abortion accompaniment and underscores the need for public policies that integrate community-based knowledge, values and practices to foster more humanised, justice-oriented models of care. *DOI: 10.1080/26410397.2026.2676395*

## Plain language summary

In Brazil, abortion is legally restricted in most cases. Even so, many women end pregnancies on their own. Feminist activists known as accompaniers support them through this process. They share information, listen, and stay present – in person or from a distance – before, during, and after the abortion. This study looks at how these activists work. We interviewed eight accompaniers in a large Brazilian city. We asked about what they do, what they know, and how they feel about the work. We found that accompaniers treat each woman as a person, not as a case to be solved. They organise the work among themselves, but when they put it into practice, they listen carefully to those they accompany, adapt their plans to each situation and to each person's needs, and combine technical knowledge with what they have learned from experience. They stay involved throughout the whole process, not just during the abortion itself. Their care is free of charge and rooted in feminist values of solidarity, autonomy, and justice. This work comes at a high personal cost. Accompaniers face exhaustion, legal risks, and invisibility. These pressures threaten their ability to keep doing the work and to reach more women who need it. Health systems and public policies should learn from accompaniers. Their knowledge and practices can help build abortion care that is more humane, more equal, and more just for everyone.

## Résumé

Cette étude qualitative examine le travail de militantes féministes qui accompagnent les avortements autogérés dans un grande agglomération urbaine du Brésil, où l’avortement reste criminalisé dans la plupart des cas. Fondée sur des entretiens approfondis avec huit accompagnatrices et s’inspirant du système brésilien de santé collective et de l’éthique féministe du soin, l’étude analyse comment les accompagnatrices intègrent technique, éthique, engagement politique et conscience sociale, transformant ainsi l’accompagnement en une véritable pratique de soin. Les résultats montrent que l’accompagnement reconnaît les personnes accompagnées comme des sujets dotés de connaissances, de désirs et de besoins évolutifs, et non comme de simples bénéficiaires passifs. Le soin est organisé collectivement, mais toujours adapté et réactif aux besoins de chaque personne, de manière interactive, critique et réflexive. Ce faisant, les accompagnatrices associent les connaissances techniques et scientifiques à l’expérience acquise grâce à des accompagnements antérieurs et aux échanges avec des réseaux de confiance. En s’engageant à toutes les étapes du processus et guidées par un projet dont le but ultime est de faire de l’avortement une expérience positive pour les personnes accompagnées, les accompagnatrices préviennent la fragmentation et l’aliénation du travail de soin. L’accompagnement apparaît donc comme un modèle de soin ancré dans des besoins radicaux: des besoins qui apparaissent au sein des conditions sociales existantes, mais qui ne peuvent y être pleinement satisfaits. Une fois reconnus et exprimés collectivement, ces besoins révèlent les limites de l’ordre établi et deviennent une source de pression en faveur d’une transformation sociale. Le coût personnel élevé de ce militantisme, marqué par la surcharge de travail, l’insécurité et l’invisibilité, compromet sa viabilité et son développement. En décrivant les éléments qui composent l’accompagnement de l’avortement, cette étude permet de mieux le comprendre et souligne la nécessité de politiques publiques qui intègrent les connaissances, les valeurs et les pratiques communautaires afin de promouvoir des modèles de soins plus humains et plus axés sur la justice.

## Resumen

Este estudio cualitativo examina el proceso laboral de activistas feministas que ofrecen acompañamiento para la autogestión del aborto en uno de los principales centros urbanos de Brasil, donde el aborto sigue siendo penalizado en la mayoría de las circunstancias. Basado en entrevistas a profundidad con ocho acompañantes, y a raíz de la salud colectiva y la ética feminista de los servicios de salud de Brasil, el estudio analiza cómo ellas integran técnica, ética, compromiso político y conciencia social para transformar el acompañamiento en una práctica genuina de atención a la salud. Los hallazgos muestran que el acompañamiento reconoce a las personas atendidas como sujetos con conocimientos, deseos y necesidades en evolución, y no como destinatarias pasivas. La atención brindada es organizada de manera colectiva, pero se adapta y responde siempre a las necesidades de cada persona, de una manera interactiva, fundamental y reflexiva. De este modo, las acompañantes combinan conocimiento técnico-científico con conocimiento experiencial adquirido mediante oportunidades anteriores de brindar atención y realizar intercambios con redes de confianza. Al participar en todas las etapas del proceso, guiadas por un proyecto cuyo propósito final es lograr que la experiencia de aborto sea una buena experiencia para las personas acompañadas, las acompañantes previenen la fragmentación y alienación de la labor de brindar atención. Por lo tanto, el acompañamiento emerge como un modelo de atención fundamentado en necesidades radicales – que no pueden satisfacerse bajo las condiciones sociales actuales, pero que, una vez reconocidas y expresadas, ejercen presión sobre el orden establecido y revelan su potencial transformador. El alto costo personal de este activismo, marcado por sobrecarga, inseguridad e invisibilidad, supone un reto para su sostenibilidad y ampliación. Al describir sus elementos constitutivos, este estudio mejora la comprensión del acompañamiento durante el aborto y subraya la necesidad de aplicar políticas públicas que integren el conocimiento comunitario, los valores y las prácticas para fomentar modelos de atención más humanizados y orientados a la justicia.

## Introduction

Abortion is a historically persistent phenomenon whose forms, meanings, and conditions are shaped by political disputes, norms, and technologies.^1^ The discovery of misoprostol in the 1980s^2^ enabled safer abortions both within and outside medical settings, and its use, alone or with mifepristone – the latter widely regarded as the global gold standard for medication abortion – is well documented as clinically safe and effective, whether provider- or self-managed.^3,4^

In the context of self-managed abortion outside formal health services, activists and non-governmental organisations (NGOs) have developed informal protocols for its safe practice since the 1990s.^5^ One form of activism that became more systematised in the 2000s, particularly in Latin America, is feminist accompaniment. In this model, feminist activists support people who need an abortion but who face barriers to access for a range of reasons, including legal restrictions. This support takes various forms: providing evidence-based information about medication abortion, helping people secure medications, and accompanying them emotionally, logistically, and clinically before, during, and after the abortion. Feminist accompaniment helps to improve abortion experiences, reduce stigma, and prevent poor health outcomes.^6^ Over time, this practice expanded through interconnected networks of activists and became what Braine^7,8^ describes as a growing transnational *autonomous health movement*, in which direct action is expressed through the provision of care. Self-managed medication abortion with accompaniment support is well documented as clinically safe and effective.^9^

Characterised by collective organisation and unrestricted access, accompaniment requires no prior affiliation of pregnant people with the movement to receive support.^7,8^ Structurally, the accompanying groups have adaptable and flexible structures that enable them to respond to diverse legal and social contexts^10,11^ and care may be delivered by telephone, online platforms, or in person.^7,8^ Members are divided into roles that include direct support, coordination, training, and administrative tasks, and some groups also emphasise collective well-being and care for the team itself.^10^

Central to accompaniment practices are the communal use of medical knowledge and technologies, reduced medicalisation, and evidence-based approaches, combined with person-centred, empathic, and trustful care that avoids criminalisation, reduces stigma, offers emotional support, and is grounded in social justice.^7,8,12–14^ Accompaniers also recognise the importance of facilitating access to health services when necessary and building selective collaborations with health professionals.^7,11^

A survey^10^ of 515 Latin American activists found that their average age is 33; most have higher education degrees and full-time jobs, and nearly half have personal abortion experience. Those in restrictive settings face greater barriers, such as limited access to abortifacients, reduced team capacity, and scarce financial resources, which affect sustainability and reach.

The literature has increasingly pointed out that the transformative potential of accompaniment remains obscured if viewed solely through the traditional lenses of public health and of legal rights that dominate the debate.^8,13,14^ The public health lens, that supports accompaniment in epidemiological terms of efficacy and safety, has been historically fundamental, influencing World Health Organization (WHO)^3^ protocols that removed the procedure from hospitals and from the exclusive control of physicians. Yet, taken in isolation, it risks reducing accompaniment to its technical-assistance dimension.^15^

The legal lens, grounded in rights-based frameworks that emphasise expanding access where the state fails, also remains limited, as it tends to frame accompaniment merely as an alternative route to the procedure in contexts where it is poorly regulated or legally obstructed. It does not explain, for instance, why even in countries where abortion is legally guaranteed, people still seek support within feminist structures either instead of, or in parallel with, formal health services, presumably because they inspire greater trust and foster more positive physical and emotional experiences.^6,16,17^

As McReynolds-Pérez^13^ and colleagues have pointed out, addressing this gap requires recognising that accompaniment is a *feminist health project* that affirms another way of caring, distinct from the hegemonic medical system, which is usually technically competent in *treatment* but ethically insufficient in *care*. According to them, this alternative way of caring is informed by philosophies of a *feminist ethics of care* and by an *intersectional consciousness.*^13^

Building on this perspective, we argue that, both in accompaniment and in any health practice, whether institutional or not, what makes it truly transformative and emancipatory lies precisely in its orientation towards care. We emphasise, therefore, that practices oriented towards care differ from those reduced to treatment by integrating ethical–political awareness and social consciousness *with* technical effectiveness in a co-produced, inseparable, and interdependent way. In the case of needing an abortion, for instance, it would be difficult for someone to describe having been truly cared for if the pregnancy was not effectively terminated. In other words, prioritising one dimension over the other inevitably obscures essential aspects of care.

To our knowledge, no previous study has undertaken this analytical articulation within the feminist project of health production embodied in accompaniment. This article aims to illuminate the elements that enable accompaniers to integrate technique, ethics, and social awareness in their practices, thereby transforming accompaniment into a genuine practice of care. Our analysis centres on the context of care production and offering within abortion accompaniment in Brazil. In doing so, we seek not only to deepen understanding of accompaniment itself, but also to inspire more integral, ethical, and emancipatory practices, both within and beyond health systems and institutions.

To this end, we draw on the health work process framework developed within Brazilian collective health *(Saúde Coletiva)*, in dialogue with the feminist ethics of care proposed by Fisher and Tronto.^18^ Before presenting these frameworks, we briefly contextualise the field in which our study is situated.

### Contextualising the field

Despite having one of the strictest abortion criminalisation regimes in Latin America,^19^ allowing termination only to save the pregnant person’s life, in cases of rape, or of fetal anencephaly,^20,21^ nearly one million procedures occur annually in Brazil, most of them clandestine.^22^ This scenario is marked by stark social inequalities where race, gender, and class unevenly affect access to information and sexual and reproductive health care.^23,24^ These same social determinants also shape access to abortion provision through feminist telehealth initiatives, as documented by Larea and colleagues.^25^ High stigma^26^ and gender-based violence worsen these barriers, intensified by far-right advances, anti-gender rhetoric, and ultraconservative policy influence.^27^

Although the exact moment when accompaniment became a recognised and organised practice in Brazil is unclear, it has been documented in recent studies, mostly over the past decade. Lauterbach^28^ focuses on accompanier narratives, stressing solidarity, sorority and the political-emotional foundations of care. A recent study^29^ also shows Brazilian feminist networks linked to NGOs and transnational groups working to expand access to safe abortion.

The scarcity of studies in the country, whether from the perspective of those providing or receiving support, reflects its clandestine nature due to legal restrictions on abortion and penalisation of misoprostol use outside authorised settings.^30^ Combined legal and health regulations limit access to safe methods and criminalise activism, reducing visibility and hindering research.

The following section provides a brief historical overview to contextualise the theoretical frameworks adopted in this study.

### The health work process and feminist ethics of care: theoretical frameworks for integrating the technical and non-technical dimensions of care

Collective health, whose historical roots lie in Latin American social medicine, constitutes an interdisciplinary and multi-professional field of knowledge and practice in Brazil, born from a critique of the medicalisation of social matters.^31^ It is marked by a political and epistemological intention to break with traditional biomedical and preventive models, seeking a broader understanding of the health-disease process and the transformation of hegemonic health practices.^32^

Since the 1970s, the field has proposed articulating epidemiology and clinical practice with the social sciences, such as sociology, anthropology, history, and philosophy, in order to restructure the health field by incorporating symbolic, ethical, and political dimensions. The adoption of Marxist and other critical perspectives was decisive for the consolidation of collective health as a school of thought, a social movement, and a theoretical practice, establishing itself as a distinct field of knowledge and action in its own right, beyond medicine or public health. It is distinguished by its understanding of health, illness, and care practices as socially determined processes.^32,33^

The context in which this perspective emerged in Brazil was marked by the curtailment of democratic freedoms, political persecution, and profound social and health inequalities. The perception that the health conditions of the population were closely related to the organisation of social life underpinned the *Reforma Sanitária* (health reform) in the country, a civil society movement linked to the struggle for democracy, which culminated in the creation of Brazil’s Unified Health System (SUS) and consolidated health as a social right and a duty of the State.^32^

The reconstruction of knowledge and practices proposed by collective health was, therefore, articulated with a broader project of social transformation. In this process, two theoretical concepts became central foundations of the health reform movement: the social determination of the health-disease process, and the health work process.^34^

The latter, developed in the 1980s by Mendes-Gonçalves,^35,36^ proposed understanding the production of health care as the result of a social work process, stripping medicine and its techno-sciences of their supposed neutrality and situating them within the world of work. From this perspective, the alienation of health professionals in relation to the ethical, political, and social determinants of their practices becomes evident, exposing alienation as one of the mechanisms through which the medicalisation of the social is reproduced. In other words, by restricting their activity to the biomedical dimension and being content with medicalisation, these subjects reproduce socially alienated practices.^37^

The health work process comprises four interrelated components: the *object*, that is, the health needs upon which one intervenes, which is always oriented by a perspective and a transformative project; the *instruments*, whether material or symbolic, which range from technologies and equipment to knowledge, practices, and forms of communication; the *purpose*, which means the intended transformation that gives meaning to work; and the *agents*, who are historically situated subjects whose actions are informed by values, knowledge, and social projects.^35,38^

Although originally developed from the analysis of physicians’ work, the concept of health work is now applied to the study of different health providers’ practices. Its analytical strength lies in its capacity to articulate structural dimensions (institutional, technological, and organisational) with the subjective and relational dimensions that shape care.^38^ Several schools of thought have deepened this framework in order to contribute to the reconstruction of inclusive and emancipatory health practices.^36^ Prominent figures of this Brazilian theoretical tradition include Lilia Blima Schraiber, José Ricardo de Mesquita Ayres, Emerson Merhy, Gastão Wagner de Sousa Campos, and Madel Luz.

According to Schraiber,^39^ the crisis in healthcare assistance is not limited to the structure of health systems but reflects a crisis in the very mode of producing care. Rooted in the institutionalisation of medicine within modern capitalism and shaped by market logic, managerial organisation, and scientific rationality, this crisis manifests in unequal access, bureaucratisation, depersonalisation of care, and deteriorating working conditions. It also involves reducing healthcare practice to a predominantly technical act centred on procedures and technologies, detached from its ethical, relational, and social dimensions.

This reduction is sustained by a societal perception that healthcare practice is merely the application of scientific protocols, expressed in uniform, predictable, and context-independent acts. At the same time, scientific knowledge needs to be reinterpreted to reflect the specificities of individual cases, giving practice an intrinsically experimental character. Within this ambiguity, professionals often neglect or devalue what they do not recognise as scientific: the social aspects of illness, the organisation of care, relationships with patients, and their own experiential knowledge.^39^

Although scientific advancement is essential for expanding therapeutic possibilities, when technological rationality becomes hegemonic, practice tends to subordinate itself to technique, distancing itself from its ethical and social purposes. Changing this rationality is difficult but necessary for transforming *treatment* practices into *care* practices.^40^

Ayres^41^ sought to analyse the relationship between the subjects involved in the core of healthcare practices to understand what differentiates treatment from care, indicating that the former is associated with achieving technical success, whereas the latter relates to practical success. Applying these concepts to everyday healthcare practices helps to clarify the options and conceptions of “good practice” at stake in health work.^40^

A response based on technical success is limited to the appropriate use of technological resources, with technique reduced to instructions on how to proceed, based on standardised protocols. It represents success in the “how to do”, that is, in correctly applying a method, instrument, or technology. A response based on practical success, in turn, refers to how the result of an action is experienced and recognised by the person cared for as satisfactory – in other words, the effectiveness of the action in lived reality. Such a response goes beyond technical correctness: it depends on whether the result makes sense to the person cared for, and it emerges from a shared project of care.^40,41^ In this sense, practical success encompasses technical success: it couples scientific and technical effectiveness with a genuine interest in making the patient's life better on the patient's own terms, rather than only producing a correct diagnosis or treatment.

This view of care from the perspective of the person cared for also appears in Fisher and Tronto’s feminist theory.^18^ In the fourth phase of care, receiving care, the authors emphasise the importance of caregivers taking into account the perceptions of care receivers when evaluating the quality of the intervention, which should be measured according to the satisfaction of their needs.

For Schraiber,^40^ three attributes converge in the professional action of healthcare workers when technical success is oriented by practical success: being interactive, critical, and reflexive. These attributes concern both how scientific knowledge is used and the practical decisions that caregivers make in their daily work.

*Being interactive:* In the field of health, human needs constitute the object upon which caregivers act. Fisher and Tronto^18^ argue that the greater the asymmetries of power between caregivers and care recipients, the more difficult it is to reach agreement on which needs should be addressed in the care process. Schraiber^40^ reflects that overcoming this obstacle partly depends on the caregiver’s willingness to interact with the person cared for. One implication of this is a shift from a persuasive stance to a dialogical agreement between the subjects. Interactivity, in this sense, does not imply erasing asymmetries, but rather exercising power ethically within unequal power relations.

By engaging dialogically, caregivers modify their actions based on what they receive from the person with whom they interact.^40^ In this interaction, caregivers recognise that the care recipient is the primary authority in establishing what counts as “good care”, that is, the values guiding what will be done and what will be considered a practical success.^41^ Ethical care, therefore, is not a predefined set of rules or fixed values but one that, through interaction, puts into practice ideas such as respect, protection, autonomy, and trust, in relation to each person being cared for.

*Being critical:* From what has been discussed, reaching the other also requires that the caregiver’s relationship with technical and scientific knowledge be critical. In the face of needs revealed through interaction, the caregiver must revisit and question the convenience of using technology, considering the limits of its pertinence. This does not mean altering scientific knowledge or the technological resource itself but rather reflecting on what to do with them, based on the new elements that arise for judging the intervention.^40^

Critical action, as proposed by Schraiber, is one that interrogates technical-scientific knowledge not to deny it, but to submit it to ethical and contextual reflection, oriented by the concrete needs of those cared for. It reorders the relationship between technique and purpose, subordinating the “how to do” to the “what to do”.^40^

*Being reflexive*: To be reflexive is to think about what one does, to revise decisions, assess effects, and re-elaborate action.^40^ In reflexive practice, caregivers think about their actions in light of previous care experiences, without reducing them to the replication of protocols or the use of scientifically adequate resources.

It is therefore necessary to consider both knowledge that derives from science, and knowledge that is constituted through experience in concrete situations – practical knowledge.^39^ Practical knowledge is ethical by nature; it integrates scientific and non-scientific dimensions and is the knowledge to which caregivers resort when facing the tensions and uncertainties of everyday practice.

Although the feminist ethics of care have been applied previously in studies on accompaniment, incorporating Fisher and Tronto’s^18^ concept of the phases of care adds further depth and contributes to bridging feminist theory with collective health scholarship. They conceptualise care as a multi-phase process – *caring about, taking care of, care-giving and care-receiving –* each involving distinct moral and practical dimensions. This framework also underscores that care practices are deeply embedded within specific social and institutional contexts, and are often shaped by inherent tensions and contradictions.

Using the contributions of collective health theory to analyse care production within feminist accompaniment practices is not meant to categorise accompaniment simply as a form of health care, nor to replace accompaniers’ own frameworks with health theories. Our aim is to expand the understanding of this autonomous movement and to learn from the accompaniers, building fruitful connections with a field that has, since its inception, resisted hegemonic medical practices.

## Method

The empirical data discussed here is part of a broader study on induced abortion, harm reduction, and care, which investigates practices both within and outside the formal health system in Brazil. The study included interviews with primary healthcare professionals as well as with feminist accompaniers. This article focuses on the findings related to feminist accompaniers, while part of the results concerning health professionals has been published elsewhere.^42^

### Study design

This exploratory qualitative study investigates the practices, meanings, and knowledge mobilised by abortion accompaniers, focusing on care dynamics in restrictive contexts. This methodological approach was chosen for its ability to access lived experiences, subjectivities, and practical rationalities, as well as the need to provide an initial overview of the issue whilst seeking to understand its meaning.^43^

### Setting and research participants

Fieldwork was conducted in a large Brazilian city. Participants were recruited using snowball sampling, starting with an activist known to the researchers. Inclusion criteria were: being involved in abortion accompaniment, being based in the city where fieldwork was conducted, and being available for an in-person interview. Despite barriers posed by criminalisation, risk, and fear during recruitment, eight interviews were conducted between October 2023 and January 2025, with informed consent and full confidentiality ensured. The final number of interviewees was determined by theoretical saturation,^44^ supported by the richness and consistency of participant narratives. Depth of analysis and the homogeneity of the participants’ activist profiles contributed to the robustness of the interpretative material.

### Data production

Interviews lasted between 47 and 120 minutes and explored motivations and feelings experienced at work, perceptions of care, daily strategies, knowledge and techniques employed, and challenges encountered. Participants were also invited to describe specific experiences, including cases they considered successful and others in which the outcomes were less desirable or which did not go as expected. All interviews were conducted by the same researcher, recorded with consent, and professionally transcribed under confidentiality. To protect the identity of participants, all interviewees were assigned identification codes consisting of the letter “A” followed by a sequential number. These codes replace participants’ names in the interview excerpts presented in the Results section, and are the only identifiers retained in the dataset.

### Authors’ positionality and ethical considerations

The authors, two Brazilian physicians and a US social scientist, are reproductive rights activists engaged in harm reduction. Our proximity to the field facilitated our understanding of the work process, but also required us to see the familiar as strange, since familiarity does not necessarily mean knowledge.^45^ In analysis, we adopted a critical and reflective stance towards our own experiences to reveal the complexity, tensions and diversity present in the accompaniers’ narratives.

This article is marked by a core tension: the need to produce and disseminate knowledge while upholding an ethical responsibility to protect the activists involved and ourselves as researchers. In some cases, we deliberately withheld details that could have enriched the analysis to avoid exposing individuals or networks. The criminalisation of abortion casts a persistent shadow over both the care provided and the research process. Rather than hindering action, however, it underscores the resilience of feminist agency: abortions continue, activist networks provide care, and ethically engaged research remains possible within this landscape of risk and resistance.

Ethical approval for this study was granted on September 21, 2023, by the Research Ethics Committee of the Hospital das Clínicas of the Faculty of Medicine, University of São Paulo (Opinion no. 6.314.710).

### Data analysis

Data were analysed using a hermeneutic-dialectic method^46^, which combines the interpretation of the meanings that participants build in their reports with a critical examination of the contradictions, tensions, and historical and structural conditions that shape these meanings. As theoretical frameworks, we draw on feminist care ethics^18^ and two dimensions of the theory of the health work process: the articulation between needs and purposes ^35,36,38^ and care interactions between subjects understood as the result of the articulation between structural aspects and ethical, technical, and emotional dimensions.^39–41^

The material produced was coded and organised into thematic categories that capture the essence of activist work. Themes were developed through both inductive and deductive processes: the first and third emerged inductively from the interviewees’ accounts, while the second emerged primarily from the theoretical framework, drawing on concepts we sought to explore in the empirical material. The themes are: (1) Collective organisation of care work; (2) The care encounter: from planned structure to practice; (3) Between commitment and fatigue: sustaining feminist accompaniment work.

## Results

Before delving into the analytical themes, we begin by outlining a basic profile of our interviewees, noting that further details about their knowledge and expertise around abortion are explored later in this section.

### Characterisation of the interviewees

All interviewees are cisgender women, most of them between 30 and 40 years old, and have supported people undergoing abortion for 1–10 years. It suggests that their involvement in activism played a significant role along their paths, indicating a close relationship between their activist experience and the shaping of their adult lives.

All eight interviewees held higher education degrees in fields such as social sciences, performing arts, history, library science, communication, and law. Three also held postgraduate degrees, with Master’s degrees in anthropology, public health, and political science. One was also a doula. This aligns with findings of high educational attainment among abortion accompaniers.^10^ Seven identified as white and one as black. Despite efforts to recruit more non-white participants, scheduling interviews proved difficult. This may reflect structural inequalities that lead racialised accompaniers to adopt stricter self-protection strategies, including avoiding participation in research involving talking about practices that are potentially under surveillance. Their under-representation also suggests that race may shape both visibility and perceived legitimacy within social movements. In contrast, whiteness^47^ and academic credentials may offer a sense of protection or social legitimacy, and facilitate engagement with activism and research.

According to Fisher and Tronto,^18^ any caring process begins with the subject or subjects *caring about* aspects of the environment that affect their survival or well-being. Therefore, *caring about* something is intimately related to knowledge: people are expected to know something when they claim to care about something.

When asked about the reasons that led them to accompany others, most of our interviewees referred both to knowing about the intense emotional suffering and isolation experienced by those seeking abortion, and to being aware of the high mortality and morbidity associated with it. Accompaniment was therefore described as a way to reconfigure this experience collectively.

“*There's the motivation to use all the knowledge that I've managed to build – and that is still under construction – on how to perform an abortion and mitigate problems and insecurities and risks and, more than that, how to produce safe experiences, experiences in which it's possible to elaborate the abortion process outside of the trauma-regret binomial, in which we can offer a multiplicity of other experiences.*” [A4]

As has been widely documented in the literature, accompaniment was also understood as part of feminist political formation, translating concepts such as reproductive justice, compulsory motherhood, gender, race, and social inequality into embodied practice.^13,48,49^

### Collective organisation of care work

Every process of transformation that arises from humanly recognised needs and is carried out with intentionality and purpose can be defined as work.^35,38^ Purpose gives meaning to action and represents the transformation one seeks to achieve and the result that guides and precedes the act itself. As expressed by our interviewees, their work aims to ensure abortion experiences that are dignified, safe, effective, and socially and emotionally supported.^13,49^ As already noted in the literature, the values that orient the way this work is carried out in order to achieve its purpose are grounded in intersectional feminism.^13^

At the time of the interviews, all participants were part of larger accompaniment groups. For some, their involvement began individually, by helping friends or people close to them, as they were seen as trusted figures within their networks – the so-called “*feminist friends.”* Later, they were invited to join organised structures or to help found them. Others joined groups directly, having been invited by people close to them or after participating in training and outreach activities aimed at mobilising new members.

“*It was really cool because the sisters from [collective name withheld for confidentiality], which I'm part of nowadays, came to do what we call a ‘Tupperware meeting’, to spread the word, to get more people to be able to work in the practice.(…) I received training on legal aspects, health aspects, safety aspects and at the end of the training, they would ask: ‘Now if you feel comfortable, you can start.*’” [A2]

In addition to sharing the same purpose, belonging to a collective also means believing that this purpose can be achieved through certain means, which members mutually agree are the most appropriate and which thus constitute their internal organisational and operational care protocols. In the process of sharing the agreements that guide care provision, they build emotional bonds with one another, centralise support requests, distribute tasks, share care demands, discuss complex cases, improve practices, recruit new members through ongoing training, and protect one another by enforcing safety rules to reduce the risk of criminalisation. It is not by chance, therefore, that these collectives illustrate what feminist scholars have described as “infrastructures of abortion care”*.*^10,50^

The collectives’ care protocols are quite similar to one another and to other models already described in the literature.^7,13,14^ They vary in some aspects according to the members’ perceptions of their technical capacity to ensure safety (for both themselves and the accompanied persons) and of how vulnerable they feel in relation to criminalisation. This reveals that it is not only the legal context that influences the model, but also its members’ profound sense of responsibility towards themselves and those they care for.

The main points of variation among the groups concern the types of cases they handle, generally determined by gestational age, and whether or not they restrict their geographic area of operation. All of them accompany first-trimester abortions within their own cities, but some avoid more advanced pregnancies due to logistical challenges and the complex emotions involved. Working outside their own city varies for two main reasons: some perceive that the criminalisation risk increases when relying on communication technologies, while others understand that distance itself imposes limitations on providing direct and high-quality care, especially in more complex cases. To address their own limitations, collectives may refer cases to one another.

Communication between accompaniers and those they accompany occurs in multiple ways, ranging from in-person contact to exchanges mediated through secure communication technologies. Accompaniers’ contacts mainly circulate between those already supported and those seeking help. Other access points include social networks, feminist collectives, NGOs, and referrals from allied healthcare professionals. The latter reveals a particularly relevant aspect of accompaniment: even those socially recognised as holders of technical-scientific authority refer patients to activist networks, implicitly recognising that these accompaniers are “lay experts”^51^ who hold valuable knowledge and provide forms of care that professionals themselves do not offer, maybe due to fear, legal and ethical constraints or limited training or experience.^42,52^

*“Our email circulates among feminist doctors, whom we often don't even know: ‘Oh, it was Dr. So-and-so’. I've never heard of that doctor in my life, but it was Dr. So-and-so.”* [A1]

Contact sharing also involves deliberate outreach to communities where there are people affected by social intersections that place them at greater risk:

*“We organise conversation circles with people who live in more peripheral areas, (…) with comrades from other social movements. We have some more direct access to sex workers too, in a more spontaneous way – because we accompanied one or another, and then, little by little, we started working through them on these multiplication points. We try to work with these groups, thinking about a strategy for each one. We try to make this work reach those for whom it could possibly make the most difference. Our interest is mainly in reaching those who have almost no options at all – to really make a bigger difference.”* [A4]

This stance shows that accompaniment collectives do not take a passive approach of merely waiting to be sought out by those in need. Instead, they proactively reach out to those who most need support and who would likely face greater obstacles in accessing them or any form of care, such as individuals experiencing poverty, irregular migration status, gender-based violence, or housing insecurity.

By acting in this way, accompaniers demonstrate a profound engagement with the social realities of the country in which they live, where abortion incidence is higher among racialised women and those with lower socio-economic status, while at the same time these groups face greater barriers to accessing safe abortion methods and post-abortion care.^23,53^ Previous research also indicates that this inequality persists in internet-based tele-abortion strategies operating in the country.^25^ In this sense, the approach described by our interviewees reflects yet another facet of their radical commitment to mitigating inequalities in access to care, a stance strongly aligned with Latin American intersectional feminism and already highlighted in the literature on the topic.^13^

Accompaniment does not operate as a transactional service.^7^ Accordingly, most collectives receive voluntary donations post-procedure, which are not a condition for care. These contributions sustain a “wheel of support,” enabling financial aid from those who can help the most vulnerable.

As Braine^8^ noted in her analysis of autonomous health movements, collectives do not require care recipients to become part of the movement. This reflects both respect for individual choice and a safety strategy, especially in contexts of high criminalisation, such as many Latin American countries, where accompaniers tend to organise in small, cohesive groups and maintain intimate circles of trust.^54^

### The care encounter: from planned structure to practice

Embedded in collectives with clear purposes, accompaniers engage in the daily routines of their work and begin interacting with the people they support. The general approach described by our interviewees aligns with previous literature.^7,10,13,14^ It unfolds in phases, beginning with digital contact and an initial conversation to share information and address concerns. After the procedure, a post-abortion follow-up is carried out to check on well-being.

The first conversation also involves assessing whether the case fits the collective’s resources and criteria, and setting initial roles and boundaries. This moment considers not only the needs of the person seeking support, but also the dynamics of the relationship being established and what the collective can realistically sustain, since in care practices both material and immaterial resources may not always be accessible.^18^

As also described previously, from the outset accompaniers emphasise a non-judgmental and person-centred approach.^14^ In practice, this means drawing on elements of active listening^55^ to genuinely understand each person’s physical, emotional, and social context. This involves starting with open-ended questions such as “How are you?” or “How is your day going?” and gradually narrowing down to more specific questions about sensitive issues that they know in advance may affect the person’s experience. Questions about gender-based violence and socioeconomic conditions were particularly prominent.

“*I ask about her context, if she's in a situation of violence. Especially from the first report, you can already tell that it's a person with a history of extreme vulnerability, whether it's social vulnerability, food insecurity, domestic violence, or not domestic violence, but gender violence*.” [A4]

Over time, they cultivate a sensitive gaze and refine their communication skills to better obtain the information they need:

“*It's difficult to ask someone: ‘Are you hungry, do you have any food at home?’ So I ask them in a different way, I say: ‘Wow, I've just had coffee. What did you eat today?’, ‘I'm so hungry, what are you having for lunch?’ And then people sometimes say: ‘Oh, I'm just having beans for lunch today’, ‘Oh, today I'm just going to have such and such’. And then you end up going into the person's home a bit, so you can understand if they need any kind of support.*” [A1]

They also have in-depth conversations about health history, medication use, and specific aspects of the ongoing pregnancy, such as signs and symptoms, and estimated gestational age.

“*Depends on what the person shares at first. And then, after they tell me, sometimes they don't have the information I need, I ask them more specific questions, related to their age, if it's their first pregnancy, if they have anyone supporting them, knowing about the pregnancy and supporting their decision to terminate, if they have any chronic illnesses, if they take continuous medication, if they've had an ultrasound or any other test, the date of their last period, all these things. Based on the answers, we move on.*” [A8]

Aiming to provide abortion experiences that are dignified, safe, effective, and socially and emotionally supported, accompaniers recognise that each encounter may bring to light unanticipated needs. These extend beyond biomedical information and *technical success*, requiring attentiveness to social, economic, and interpersonal elements that may lead to *practical success.*^40,41^ They identify these needs by engaging interactively and opening themselves to the encounter.

This openness, however, can sometimes trigger scepticism. Some accompaniers reported that it is uncommon for people to encounter someone so genuinely interested in them:

“*The poorer girls and women, or those who live in more peripheral regions or who are in a more vulnerable situation, they start to suspect why we're building this excess of care, without profit, they start to think it's going to be some kind of scam to take their organs, organ trafficking, something like that. They don't believe it's possible to access this care, they think it's all too new, too rare for them, they're not used to people asking how (they) are feeling, what they need.*” [A4]

Other elements of initial estrangement arise from the systematic use of anonymity, as accompaniers use code names to separate personal life from activism, and from the fact that this form of care operates outside market logic, where accountability is tied to payment and care is expected as a service.^56^ The absence of financial expectations in exchange for care, according to some, stands out particularly among structurally disadvantaged individuals:

“*A girl once said: ‘Why are you helping me so much? What's your interest in this? I'm scared of you.’ Because we offered the ultrasound, we offered to accompany her to the ultrasound and all this for free. And she got scared.*” [A1]

What emerges, in practice, is that such experiences often challenge expectations shaped by prior encounters with instrumental or commodified relationships, since free, value-driven care can feel radically new and initially disconcerting. Paradoxically, what most strengthens this practice – its generosity and political commitment – may at first be perceived as a barrier, eliciting suspicion, as if the care offered were “too much” or “too generous to be real.”

This initial mistrust often requires accompaniers to clarify the nature and purpose of their work in a pedagogical yet firm way, setting clear boundaries to prevent misunderstandings about their role and motivations:

“*I’m pretty tough. Right at the start I always get serious and tell them our collective isn’t for profit, that what we do is feminist accompaniment (…) I think the definition of roles is really clear from the start: who we are, what we’re doing, why we do it.*” [A6]

Remote care, mediated through secure communication technologies, most often text-based, was also mentioned as a challenge for establishing trust and emotional connection. To overcome these barriers, accompaniers maintain consistent and agile communication, and some resort to audio or video calls to create a sense of closeness. Although communication modes are collectively agreed upon according to each group’s assessment of digital security, participants noted that such agreements can be revised on a case-by-case basis when they are perceived as affecting the quality of the relationship.

As one participant explains, having realised in practice the limits of exclusive digital mediation, she chooses to go against her collective agreements, which recommend only digital contact, and dares to pursue in-person encounters when she perceives them necessary to meet the needs of the accompanied person:

“*The safest way [in terms of avoiding legal risk] is not the most affectionate way. (..)Sometimes it also involves giving the person a hug, telling them that everything is fine, you know.*” [A2]

As widely highlighted in the literature, accompaniment embodiment^48^ plays a fundamental role in care:

“*The person isn't expecting an ordinary woman. And they say: ‘Wow, I was expecting something else. (…) I was expecting someone who looked like a drug dealer’. Then I ask: ‘But what would a drug dealer look like?’ I think putting your body on the line also changes this idea about what's involved.*” [A8]

By showing an ordinary body similar to those they accompany, accompaniers challenge the criminalised image of abortion and reframe accompaniment as an act of solidarity and shared humanity. In Brazil, the act of “putting your body on the line” takes on particular significance because no region is free from the presence of criminal organisations,^57^ and clandestinity is often linked to violence and fear.

Digital communication limitations have been previously noted in the literature. Evaluations of remote services often recommend more immediate and personal communication, such as phone or in-person contact, reinforcing the importance of presence in perceived care quality.^58^ In addition, as shown in the study on a telehealth service in Brazil,^25^ predominantly internet-mediated communication can be a mechanism through which geographic and economic inequalities in access to care are reproduced, reinforcing inequities by privileging white, wealthier, and higher-educated individuals.

All accompaniers report flexibility in care despite clear previous collective agreements, adopting personalised approaches and adjusting care according to the specifics of each case. This reflects a reflexive attitude towards care, in which decisions are guided not only by established norms but also by the continuous assessment of each person’s needs and by the capacity to question their own protocols. McReynolds-Pérez and colleagues^13^ have also highlighted the reflexive nature of accompaniment practices, emphasising this movement of going into the field, confronting diverse realities, returning to oneself, and adjusting practices in light of these encounters.

Accompaniers recognise that initial fear and distrust can be mutual, sometimes prompting them to adopt verification strategies to confirm the identity of those seeking accompaniment. As mutual trust gradually deepens through interactive exchanges, accompaniers come to understand each person in a broader and contextualised way. Through this process, a relationship grounded in trust, attention, and empathy is consolidated, allowing them to conduct a situational analysis of the lived reality of the person being accompanied.

Having gathered all the information to identify the needs at stake in the encounter, accompaniers evaluate whether they have the resources and safety conditions to proceed with their work. Resources include both material and immaterial instruments, which must be combined in unique ways to meet the specific needs that each person and situation present.^35^ Safety conditions, in turn, involve the assessment of physical and legal risks.

When the initial combination of health, material, social, and safety conditions presents an obstacle or limitation that accompaniers consider possible to overcome, they organise themselves to solve it:

“*We spent a week working on this person's diet, because she was also very anaemic. We had to look at treatment for anaemia.*” [A4]

“*A lot of precarious people arrive, we have a really high financial expense with ultrasounds, transportation, eventually cell phone credit and food*.” [A1]

“*Sometimes, there’s no access to a phone that isn’t monitored, like using a phone that she shares with her partner and can’t talk to him about it. One of the most extreme cases was supporting someone who was an undocumented immigrant in the country, in a situation of labour that was basically slavery. She didn’t have a cellphone, but she could access an email account from a computer at her workplace. She would communicate with us by email, and we had to figure out an entire plan – including how to get her out of that factory, which was under surveillance by very dangerous people. So, we came up with this whole scheme to pick her up. We arranged a meeting point by email, and then we passed her from car to car, from person to person, just in case we needed to throw anyone off. And we took her to a safe place where she could stay for a week, so we could accompany her throughout the entire process.*” [A4]

All interviewees reported similarly complex cases. These accounts highlight how accompaniers integrate social dimensions into care not as secondary considerations, but as determinants that directly shape how abortion will be experienced by those they accompany, and therefore require action on their part.

The procedure is scheduled according to availability and what works for both the accompanier and the accompanied person. Similarly to what has been observed in the use of a telehealth abortion service in Brazil,^25^ accompaniers report that most of the cases they support involve people in the first trimester of pregnancy. While these conditions often allow for remote support, some cases are more complex and require closer accompaniment. No one is left without care, so if an accompanier identifies a technical or contextual limitation in managing a case, it may be transferred to a colleague within the same collective or between local and national collectives.

Only one of the interviewees reported that the collective she belongs to prefers to hold pre-abortion group information sessions rather than individual conversations. Group meetings are a well-known strategy and have been associated with positive feelings among accompanied persons, who report feeling less isolated, sharing doubts, and normalising their experiences.^59^ The accompanier who conducts these sessions reported that, from a logistical standpoint, this strategy also benefits the accompaniers, as holding multiple small in-person meetings demands considerably more time and energy. Synchronising the guidance for several people at once, on the other hand, can even encourage the creation of virtual groups among the accompanied persons, who may support one another and share experiences in the days leading up to and during the procedure.

In both individual and group meetings, accompaniers provide detailed guidance on the steps of the abortion process, what to expect during and after it, possible side effects, warning signs that require professional care, and how to prevent criminalisation. This information can also be accessed through complementary materials such as texts, podcasts, films, booklets, and books. These materials serve to translate scientific and technical knowledge into accessible and user-friendly language, empower decision-making, and prepare the person for the experience. This anticipatory guidance, akin to clinical “safety netting,” helps reduce anxiety and unnecessary emergency visits by fostering self-monitoring.^60^ In abortion experiences, receiving clear and comprehensible information is described as one of the key elements of high-quality interpersonal care.^59^

People are encouraged to involve a trusted person during the procedure to assist with food, comfort, and emergency care:

“*We tell her that she needs someone close by, especially because when she’s in pain, it feels like she’s going to die – it’s too much. And if you’re alone, you might faint; you need someone to look after you. Make you food, stay close, give you a massage, warm you up with water. It could be a friend, a mother – whoever you want. Someone to take you to the hospital if you need it.*” [A3]

They also give specific guidance to the chosen support person, reinforcing their role in the abortion process:

“*I ask for the person's name and then I record an audio for that person, who also has a very important role: ‘Don't keep asking So-and-so questions. If you want, you can ask me anything – let's try to make their day. It should always be like that, but today in particular, let’s really take care of this person. Make them the food they like – that’s important.’ I do it to give them a boost, to remind them that it’s a day of care, and that this process is a process of care.*” [A4]

To some extent, all interviewees expressed scepticism towards male involvement in care. They reported greater resistance to men, particularly during initial contact, while female friends or relatives were generally perceived as more capable. This stems from past experiences in which male partners were perceived as controlling or had taken actions that placed either the accompanier or the accompanied person at risk. Still, this pessimistic view raises tensions, as what is perceived as control by the accompanier may be experienced as care by the accompanied person, and it also risks reinforcing the notion that men are unfit to care, an idea that much of feminism has historically sought to challenge.^18^

Abortions are performed using either misoprostol alone or in combination with mifepristone. Officially, the former is restricted to use in accredited hospitals, while the latter is not registered with the Brazilian Health Regulatory Agency (Anvisa) and therefore is not available for sale in the country. Regarding guidance on the use of medication abortion pills, the WHO^3^ protocol constitutes the main reference used by accompaniers to provide information and support to those they accompany.

The criminalisation of misoprostol in the name of public health^19^ creates a particular scenario in Brazil, with effects that extend far beyond the difficulty of accessing the medication. In the media, the drug is frequently framed in police reports related to the dangerous illegal market,^61^ rarely being presented as the safe and essential medication it is, as recognised by the WHO.

Although it has long been the most commonly used method for abortion in Brazil,^62^ its use is permeated by fear and misinformation,^63^ which requires accompaniers to include conversations that address the legal, technical-scientific, and symbolic dimensions surrounding the drug. In addition, accompaniers must deal with the fact that not all accompanied individuals have access to the dosage of misoprostol recommended by the WHO protocol. A critical, responsible, and shared adaptation of the protocol may therefore be required in certain care situations, as has already been observed in studies with other groups.^64^

Thus, accompaniers’ engagement with biomedical technologies and scientific knowledge is not distinct from, nor does it outweigh, the personal or contextual challenges they navigate in their everyday practice.

“*As far as the specific health protocol is concerned, we base ourselves on the WHO protocol, but we also listen to our partner physicians, the Latin American partner collectives, and then the [the name of an international feminist organisation removed for safety] as priority partners. (…)We have this adaptability with the concrete reality of our collective as well.*” [A6]

This situated approach, which takes into account the *imperfect conditions*^65^ of the context to deal with the “technical-scientific moment” of accompaniment – that is, the dimension concerned with the clinical-procedural aspects of medication abortion, whose procedures are defined in manuals and reflect the outcomes of discussions guided by scientific rationality – has already been documented in other analyses.^64^ Prandini^66^ has identified that this adaptability is one of the fundamental principles of accompaniment.

Accompaniers mentioned that their main networks of trust for learning, case discussions, and referrals were primarily other accompaniers. The establishment of alliances with allied healthcare professionals, already explored in previous studies,^14,67^ varied across accounts. When present, these alliances generally involved physicians, whose most frequent contributions occurred remotely. Their input included providing opinions on specific cases, such as doubts about possible drug interactions between misoprostol and medications used continuously by the accompanied person, reviewing complementary exams, or offering second opinions on guidance received from health services. They were also mentioned as trusted contacts to whom accompanied persons could be referred when they wished to initiate long-acting contraceptive methods after the abortion.

Practical experience, the challenges it entails, and the ongoing dialogue with accompanied persons, other accompaniers, health professionals, and various actors directly or indirectly involved in care all nurture a form of experiential knowledge that connects to the notion of practical knowledge.^41^ This knowledge is then reflexively applied in future care, producing a spiral of continuously improving practice.

All interviewees considered medication abortion safe and expressed confidence in its use. They reported little or no fear of physical complications, but greater concern about legal security. While acknowledging that safety does not imply the complete absence of complications (rare, but mentioned by some), all stated that they were prepared to manage issues or refer to health services if needed, always ensuring that a safety plan is in place before the procedure begins.

Accompaniers make contact with the accompanied persons after the procedure. However, they report diverse attitudes of the accompanied persons towards this post-abortion moment. Some choose to end the relationship quickly, which accompaniers interpret as motivated by a desire to forget the experience or to return to normal everyday life. This may range from a brief, superficial reply to the accompanier’s contact to complete silence. The absence of a response is generally interpreted as a sign that the procedure has been successful. Some accompaniers also recognise that demand is so high that, if a person fails to respond, there is neither the time nor the operational capacity to carry out post-procedure checks more emphatically or systematically. Others, however, maintain longer-term bonds, returning to clarify doubts, share test results, express gratitude, refer others, or express emotional issues, moments when accompaniers provide extended listening and support.

Accompaniers reported different ways of confirming that the abortion had been completed. Most relied on physical indicators, such as the regression of pregnancy symptoms in the days after the procedure. Some recommended an ultrasound when feasible, but acknowledged the economic and logistical barriers to accessing it, as well as the fear of seeking formal health services given the role that some professionals play in abortion-related criminalisation in Brazil.

Only one participant reported that the collective she is part of actively seeks to identify and refer cases that fall within the legal grounds for abortion in Brazil to reference hospitals. This stance reflects an understanding that, despite the State’s limited engagement, it should nonetheless be held accountable for guaranteeing access to care in cases explicitly protected by law. For most accompaniers, however, formal health services were described as a last resort, an option considered only when, even in collaboration with other activists or allied professionals, they assessed that it would not be possible to ensure adequate or safe care within any collective network.

The State's presence is deeply felt by the accompaniers, both through the barriers deliberately imposed on abortion care and through the reliance on public services such as health and social services that may become necessary at different stages of the process. In such cases, activists not only make referrals but also mobilise, negotiate, and coordinate access. This remains an important dimension of accompaniment practices to be further explored in future work.

### Between commitment and fatigue: sustaining feminist accompaniment work

As Fisher and Tronto argue, all human activities involve a dimension of care because, in addition to acting, we must also sustain ourselves as actors.^18^ In the context of accompaniment, a constant challenge faced by activists is balancing their engagement with the practical and political demands of care while also caring for themselves.

Providing feminist support for abortion requires emotional availability and time, but this solidarity-based work coexists with accompaniers’ paid jobs and additional care responsibilities they need to assume, often within their own families. They highlight task accumulation and overlapping work hours:

“*This is an extra shift, so you have to think that people work, they have families, sometimes kids, and they still do this – so it’s one more shift, on top of the many shifts women already have.*” [A1]

“*Since I’m also self-employed, sometimes I don’t have anything planned for a certain day and then, all of a sudden, a job comes up. And because I have precarious labor, I can’t turn it down – especially since what we do here is volunteer work, so it doesn’t pay for that job. So I also have to negotiate with the person, like: ‘Look, I won’t be able to be there exactly how I thought I would, but from this time to that time I won’t be around, and after that I’ll be able to check in again.’*” [A2]

“*Sometimes the girls go through it at dawn, you know? And then it drives me crazy. Because, like I said, I have a kid and he wakes up super early – six o’clock, six-thirty at the latest. And sometimes I have to leave my phone on so that when she sends a message, I’ll wake up and reply. That’s also really hard for me.*” [A5]

These accounts highlight the personal cost of sustaining accompaniment work in a context marked by the advance of the neoliberal agenda, in which the effects of labour precarisation and the privatisation of care responsibilities weigh particularly heavily on women’s lives.^68^ In this scenario, accompaniment involves a constant negotiation by activists between the demands of their own lives, their collectives, and the people they support.

The heavy reliance on online communication tools adds complexity to this context. While it facilitates contact, it also makes activists fully permeable to care demands, meaning routine activities – such as rest, family life and emotional relationships – can often be interrupted by accompaniment demands, deepening physical and emotional exhaustion.

“*Overall, I feel really overwhelmed – that’s the main thing right now. More than any emotional reward or the feeling that I’m doing something necessary, it’s really the overload that stands out. I think it has to do with the fact that we do this through our phones, and we already do so many other things on our phones – we already work, we organize gigs through here. We also have fun on here, we talk to people we care about on here, some of whom we only talk to through here. So I think it ends up creating this … you know? You just feel like you’re overflowing*.” [A2]

Fast communication and expectations of instant replies, intensified by abortion-related anxiety, impose a relentless work pace. In this sense, hyperconnectivity contributes to a particular form of exhaustion, characterised by difficulty in disconnecting from care work without feeling guilty.

There is an added layer of tension linked to the degree of both social and legal criminalisation of abortion and the criminalised status of misoprostol in the country. This compels some accompaniers to keep their work secret, even from close family members, friends, and romantic partners, which makes their routines more complex, affects their mental health, and in some cases leads to social isolation. On this personal and intimate impact, one accompanier shared:

“*What really wears you out isn’t the support itself. What wears you out is the fear. I think there comes a time when you just get a little tired of being afraid, you know?*” [A7]

Combined with stress and the absence of support networks with whom they can fully share their experiences and anxieties, this feeling can lead to profound exhaustion. One accompanier reflected that this overload can affect what she considers to be the best possible care:

“*Sometimes, when we get a bit overwhelmed with accompaniments, it’s common for us to start modifying the way we do things – even becoming a bit less attentive to certain details. And sometimes those details aren’t super decisive, but they’re things you would’ve been more careful about if you had more time to focus on just one person or maybe two people at a time.*” [A1]

While this study did not aim to quantify the number of accompaniments carried out by activists and the collectives to which they belong, it would be remiss not to acknowledge the deep connection between the high demand for this type of care and the feeling of overload experienced by many of them. Activists report carrying out between one and two thousand accompaniments per year in each collective, with each activist supporting ten to thirty individuals monthly.

These figures are striking not only for the workload but also for implicitly reinforcing the urgent demand for humane abortion care. It also highlights the well-known fact that abortion is a common, everyday reproductive event, regardless of criminal law.

Some activists express a desire to temporarily step away from this activity, or mention periods when they have taken time off. However, even these moments are marked by guilt or discomfort, either for interrupting essential work or for overburdening their peers.

Despite all the difficulties, the activists reported positive feelings linked to accompaniment, as other studies have shown.^69^

“*What makes it most worthwhile is that this struggle, this work we do, brings immediate results. So it’s not like we’re planting a seed now to maybe see some big social change in I don't know how many years – it’s about what we’re building. This change and transformation, we see it every day, in every follow-up, in every conversation we have with people. I think that’s what keeps us going.*” [A8]

If abortion is not an abstraction for those who undergo it, but rather intimately tied to the concrete conditions of their life,^32^ the same applies to those who offer support. This underscores the need for accompaniers to develop both individual and collective self-care strategies within the context of activism. We identified mutual support strategies, including exchanges across national and international collectives, collaborative follow-up, scheduled rest (e.g. vacations, rotating shifts), and protection for those who are socially marked as more at risk of criminalisation.

These include, for example, shielding those engaged in other forms of confrontational activism or racialised activists from higher-risk tasks, acknowledging that certain social markers^70^ can carry specific implications for criminalisation. While a deeper investigation of these dynamics was not the aim of this study, we consider this a promising area of research within the feminist movement.

## Discussion

The purpose of accompaniment, as evidenced in this and other analyses, is to promote abortion experiences that are effective, safe, dignified, respectful, and socially supported. While effectiveness and safety are technical concepts that contain a concrete and measurable dimension tied to public health rationality, dignity, respect and social support refer to values and meanings that are constructed and redefined in each care encounter, dimensions historically claimed by Brazil’s collective health field as fundamental to the production of health.

The notion of practical success concerns the outcomes of care interventions that make sense in the lived experience of those who receive them. Practical success encompasses technical success (effectiveness and safety) while extending it to a broader dimension that includes respect, dignity, and social support, integrating the ethical and social meaning of caring. Achieving this requires caregivers to articulate instrumental and communicative actions, simultaneously oriented towards achieving an end and reaching mutual understanding.^71^

Aware of the transformative potential of feminist accompaniment and of the evidence produced so far about this movement, we analysed these activist practices through the narratives of our interviewees regarding their encounters with the people they care for, drawing on our position as physicians and activists committed to transforming health practices. Our aim was to illuminate the elements that enable accompaniers to integrate technique, ethics, and social awareness in their practices, something we understand as both transformative and emancipatory within the field of care, with potential to inspire all caregivers, formal or informal, activists, physicians, nurses, and other health professionals, despite the significant differences in how care systems are organised.

Unlike much of institutional health care, where recipients are often treated as passive objects, accompaniment recognises them as subjects with knowledge, desires, and evolving needs. As Heller^72^ proposes, human needs are neither fixed nor universal but socially produced and historically shaped, even if uniquely expressed by each person. This requires recognising care recipients as knowledgeable subjects whose desires and needs evolve throughout the process, as initial needs are met and new ones emerge.

To access those they accompany in their full complexity, accompaniers adopt an intersubjective relational approach. Their testimonies reveal that communication is valued and understood as vital to the practice of accompaniment. Through communication, they seek to grasp the complexities of each situation, extending beyond the individual to include trusted relationships and broader community networks.

Interpersonal interactions always unfold within broader social contexts that inherently carry dimensions of power. Fisher and Tronto^18^ argue that community-based spaces are less shaped by power asymmetries, which thereby enhances responsiveness to complex needs. However, as previously discussed in other research, feminist accompaniers are lay experts^51^ in medication abortion, possessing practical knowledge that is likely greater than that of most people in society, at least in the Brazilian context. This expertise positions them as authorities on the subject, often recognised by healthcare professionals who refer individuals to them.

By placing inter-subjectivity at the centre of care, accompaniers demonstrate how power can be exercised ethically rather than coercively or persuasively.^40^ They make ethical use of their knowledge, acknowledging the accompanied person’s self-knowledge as valuable for care. Care plans collectively agreed upon within accompaniment groups are therefore subordinated to the needs of the accompanied persons, not the reverse. Although grounded in collective agreements, care is tailored to each individual case. Continuously reinvented, these care plans are designed to function not only under adverse conditions but in the service of those receiving care. Each encounter thus promotes individual and collective reflection on the collective structures previously established.

This leads us to understand accompaniment as a model of care grounded in radical needs. Heller^72^ defines radical needs as those that cannot be met under existing social conditions but that, once recognised and articulated, exert pressure on the established order and reveal its transformative potential. In accompaniment, the call for dignity, respect, and social support expresses these unmet needs and affirms a collective aspiration to transform the very conditions that produce their denial. In this sense, intersectional feminist values simultaneously operate as the standpoint of care agents, the instruments guiding their practice, and the ultimate purpose of their actions.

Protocols such as those issued by the WHO are part of the technical-scientific knowledge used by accompaniers to provide care. However, just as the structure of their work is constantly subjected to reflexive adjustment to better meet people’s needs, accompaniers do not apply technical protocols mechanically. Rather, they critically combine scientific knowledge with the experiential knowledge developed through prior care experiences and exchanges within their trusted networks. The critique of biomedical rationality thus also involves valuing practical knowledge^39^ developed through intersubjective relationships and shaped by processes of reflection, critique, and exchange.

By actively participating in all phases of care,^18^ accompaniers prevent fragmentation, sustaining reflective and adaptive practices grounded in listening, critique, and reinvention. Although collectives establish divisions of tasks, accompaniment requires that accompaniers remain highly available, offering continuous communication and following each person from the beginning to the end of the process. Rejecting market logic, often absorbing care-related costs themselves, they provide care in a landscape where safe, effective, dignified, respectful, and socially supported abortion remains the exception. Yet this is an especially complex and demanding task.

Direct action, however, comes at a price. Accompaniers are rarely recipients of care themselves. Their work unfolds amid material insecurity, legal risk, scarce resources, and emotional and physical strain. Long working hours, combining paid and unpaid labour, often overlap with family responsibilities and emotional life, revealing both the personal costs of sustaining this work and the strength of their political commitment. While some self-care strategies were identified, further exploration of these practices remains a promising area for future research.

Another limitation is that this model of care, despite its high quality, cannot reach all those in need, not only because of the high personal costs for those who perform it voluntarily, but also because of the magnitude of demand. Nevertheless, by proving that such care is possible, accompaniers can serve as an inspiration for professionals and health systems. We believe that humanised care can be developed within institutional structures to guarantee health and rights for all. This perspective underpins Brazil’s collective health field, in which the public system is grounded in universality, comprehensiveness, equity, and social participation.

This study also reinforces that abortion care can and should be shared with professionals beyond physicians, as well as with non-professionals, and underscores the need for public policies and health systems to decentralise authority and incorporate the knowledge and perspectives generated through community-based actions.

### Limitations

Although this research is grounded in the Brazilian context, the tensions identified between emotional bonding, legal risk, and organisational sustainability resonate with other global settings marked by rising conservatism and restrictions on reproductive rights. The study is limited by the absence of abortion seekers’ direct perspectives and its focus on collectives based in a large urban centre, which may constrain the generalisability of findings. Including these voices in future research, as well as exploring activists’ self-care strategies, could deepen our understanding of this essential yet often invisible work in health and reproductive justice, while supporting collectives in developing sustainability strategies.

We also acknowledge that the predominance of white, highly educated participants limited access to the experiences of racialised, low-income, non-binary, and trans activists. This limitation underscores the need for future studies employing intentional outreach to include more diverse voices, particularly those operating outside major urban centres, to capture a more comprehensive view of care practices within the movement.

## Conclusion

Accompaniment constitutes an autonomous health movement whose direct form of action is providing care to those who need an abortion. Its practices are organised and operationalised through a set of material and symbolic instruments, articulated in an intersubjective, critical, and reflexive manner, aimed at responding to radical social needs and fostering the practical success of the abortion experience. In doing so, accompaniers remain aware of the entire work process, engaged in its purpose, and fully accompanying the cared-for person, thereby constituting a form of care work that is not alienated from society nor from itself.

## Data Availability

The data that support the findings of this study cannot be shared in order to protect the privacy and confidentiality of participants. The interviews address sensitive topics, and public disclosure of the data could compromise participant anonymity and the integrity of the research.
